# Developmental trajectory of the endocannabinoid system in human dorsolateral prefrontal cortex

**DOI:** 10.1186/1471-2202-13-87

**Published:** 2012-07-24

**Authors:** Leonora E Long, Jonna Lind, Maree Webster, Cynthia Shannon Weickert

**Affiliations:** 1 Schizophrenia Research Institute, Darlinghurst, NSW, 2010, Australia; 2 Neuroscience Research Australia, Barker St, Randwick, NSW, 2031, Australia; 3 University of New South Wales, Sydney, NSW, 2031, Australia; 4 Karolinska Institutet, Stockholm, Sweden; 5 Stanley Medical Research Institute, Rockville, MD, 20815, USA

**Keywords:** CB_1_ receptor, Development, Endocannabinoid, Inhibitory interneuron

## Abstract

**Background:**

Endocannabinoids provide control over cortical neurotransmission. We investigated the developmental expression of key genes in the endocannabinoid system across human postnatal life and determined whether they correspond to the development of markers for inhibitory interneurons, which shape cortical development. We used microarray with qPCR validation and *in situ* hybridisation to quantify mRNA for the central endocannabinoid receptor CB_1_R, endocannabinoid synthetic enzymes (DAGLα for 2-arachidonylglycerol [2-AG] and NAPE-PLD for anandamide), and inactivating enzymes (MGL and ABHD6 for 2-AG and FAAH for anandamide) in human dorsolateral prefrontal cortex (39 days - 49 years).

**Results:**

CB_1_R mRNA decreases until adulthood, particularly in layer II, after peaking between neonates and toddlers. DAGLα mRNA expression is lowest in early life and adulthood, peaking between school age and young adulthood. MGL expression declines after peaking in infancy, while ABHD6 increases from neonatal age. NAPE-PLD and FAAH expression increase steadily after infancy, peaking in adulthood.

**Conclusions:**

Stronger endocannabinoid regulation of presynaptic neurotransmission in both supragranular and infragranular cortical layers as indexed through higher CB_1_R mRNA may occur within the first few years of human life. After adolescence, higher mRNA levels of the anandamide synthetic and inactivating enzymes NAPE-PLD and FAAH suggest that a late developmental switch may occur where anandamide is more strongly regulated after adolescence than earlier in life. Thus, expression of key genes in the endocannabinoid system changes with maturation of cortical function.

## Background

The output of principal cortical neurons is regulated by a network of inhibitory interneurons [1,2]. Coordinated development of these neurons underlies the acquisition and adaptation of visual, motor and cognitive function throughout postnatal life. Cognitive development during critical periods such as toddlerhood and adolescence is characterised by rapid acquisition of executive functions such as language, goal-directed behaviour and working memory [3-5]. This development is subserved by processes that facilitate a balance between excitatory and inhibitory transmission such as synaptogenesis [6-8], maturation of inhibitory interneurons [9], development of GABA receptors [10-12] and an early-life switch from excitatory to inhibitory actions of GABA [13-15].

The endogenous cannabinoid (endocannabinoid) system modulates excitatory-inhibitory balance by mediating short- and long-term synaptic plasticity. Cannabinoid CB_1_ receptors (CB_1_R) are thought to be primarily localised to presynaptic terminals of inhibitory GABAergic interneurons that express the neuropeptide cholecystokinin (CCK) and target the soma or proximal dendrite of pyramidal neurons in the primate dorsolateral prefrontal cortex (DLPFC) and primate and rodent hippocampus [16-18]. In addition, CB_1_Rs have been shown to co-localise with the calcium-binding proteins calbindin and calretinin in distinct subsets of cortical inhibitory interneurons [16,18-20]. CB_1_Rs are also localised to some principal neurons in the hippocampus and the cerebellar cortex [21-23], although the current consensus is that they are expressed at much lower levels on these cells than in inhibitory interneurons [24]. While endocannabinoids signal via a number of other receptors in addition to CB_1_[25], the major mode by which they interact with CB_1_Rs is via retrograde signalling. Following membrane depolarisation, increased Ca^2+^ concentration or metabotropic glutamate receptor activation, endocannabinoid ligands are synthesised in the postsynaptic terminal. Postsynaptic release and retrograde diffusion of endocannabinoids results in presynaptic CB_1_R activation and suppression of neurotransmitter release from presynaptic [20,26-31] and, indirectly, from postsynaptic terminals [32].

Biosynthesis of one major endocannabinoid, anandamide, from membrane phospholipid precursors is catalysed by several enzymes. The most well-studied of these, which we measured the encoding mRNA for here, is N-acylphosphatidylethanolamine-specific phospholipase D (NAPE-PLD; [33,34]), but others include glycerophosphodiesterase (GDE1) [35], abhydrolase domain 4 (ABHD4) [36] and the phosphatase PTPN22 [37]. Formation of the other major endocannabinoid, 2-arachidonylglycerol (2-AG), is catalysed by alpha and beta diacylglycerol lipases (DAGL) [38]. Fatty acid amide hydrolase (FAAH) catalyses postsynaptic anandamide breakdown [39,40] while 2-AG activity is terminated by both presynaptic monoglyceride lipase (MGL) and the postsynaptic abhydrolase domain 6 enzyme (ABHD6) [41-45]. Together, these elements of the endocannabinoid signalling system regulate early nervous system development (i.e. neuroblast migration [46], pyramidal cell specification [47], interneuron migration [48] and axon guidance [49-51]) and synaptic plasticity [29,52], and thus would also be expected to sculpt behavioural and cognitive development.

It is thus critical to determine the normal developmental trajectory of the endocannabinoid system, in humans and to determine which elements may be changing during critical periods such as adolescence, when the majority of cannabis use commences and which may be a period of vulnerability to the detrimental effects of cannabis exposure [53,54]. Indeed, earlier age of cannabis use onset is associated with higher risk of psychotic symptoms and neuroanatomical abnormalities associated with psychosis [55-58]. However, while brain region-dependent increases and decreases in CB_1_R binding density and distribution between prenatal, infant and adult humans have been described [59-62], detailed studies of human endocannabinoid system development, particularly in the frontal cortex, are lacking. The aim of the present study was therefore to extend these earlier findings, using gene expression analysis to describe the development of CB_1_R and some of the major endocannabinoid metabolic enzyme mRNAs in human DLPFC across postnatal life. Given the localisation of CB_1_Rs to CCK-, calbindin- and calretinin-positive interneurons, we hypothesised that the trajectory of cortical CB_1_R development would partially correspond to those previously determined for these interneuron marker mRNAs in the same subjects [9].

## Results

Expression of housekeeping gene mRNAs did not change significantly over the lifespan, nor did the geometric mean of the expression for all four housekeeping genes (Additional file 1: Figure S1).

### CB_1_R mRNA decreases across postnatal life

**Figure 1:**
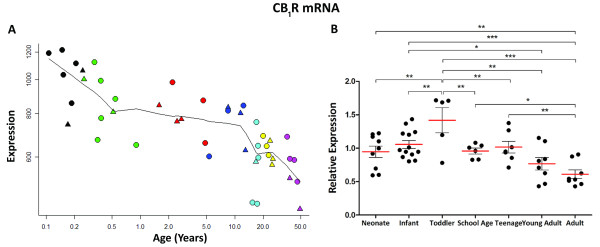
**CB**_**1**_**R mRNA in developing human DLPFC determined by (A) microarray (y-axis, arbitrary units in log scale) across age (x- axis, log years) and (B) qPCR [y-axis, mean (+ S.E.M.) expression normalised to the geometric mean of four housekeeping genes] plotted by age group.** * P <0.05, ** P <0.01, *** P <0.001 (ANCOVA and post-hoc LSD).

Microarray analysis revealed a significant decrease of approximately 50% in CB_1_R mRNA (probeset 213436_at) across the human lifespan (r = −0.779, p < 0.001, Figure 1A). Expression decreased from a peak in neonates to a plateau from toddler age until adolescence, and then further decreased to adult levels. qPCR data showed that CB_1_R mRNA decreased across postnatal life and correlated with RIN (r = 0.458, p < 0.001) and PMI (r = .337, p < 0.01) (ANCOVA, F = 5.5, df = 6, 47, p < 0.001, Figure 1B). Post-hoc LSD tests showed that CB_1_R mRNA peaked in toddlers and was lower in the adult group than in all other earlier age groups except young adults (see Figure legend for other group comparisons).

**Figure 2:**
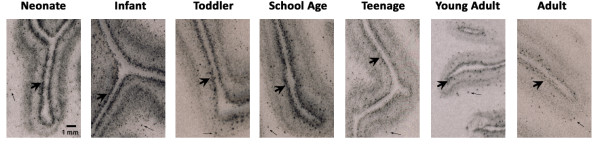
**Representative autoradiograms of CB**_**1**_**R mRNA hybridisation in each age group of the developing human DLPFC.** Large arrowheads: CB_1_R mRNA signal in layer II; small arrows: scattered signal in white matter.

**Figure 3:**
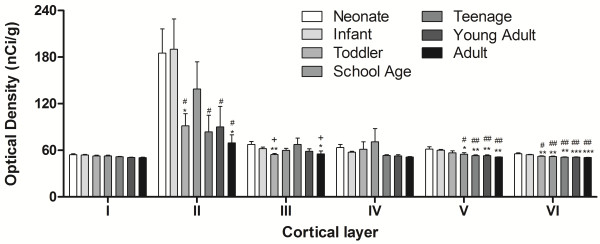
**Mean CB**_**1**_**R mRNA expression in each layer (I – VI) of the developing human DLPFC.** Data represent mean (+ S.E.M.). n = 5 – 13. * P <0.05, ** P <0.01, *** P <0.001 vs. neonate, # P <0.05, ## P <0.01 vs. infant, ^ P <0.05 vs. teenage (post-hoc LSD).

**Figure 4:**
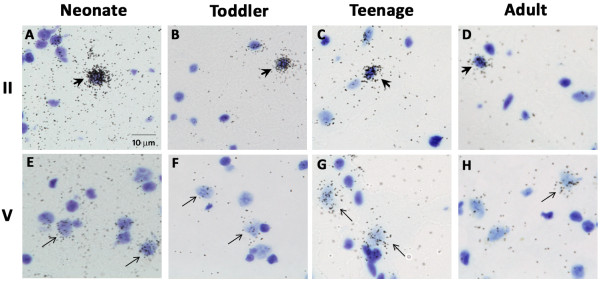
**High power brightfield photomicrographs of CB**_**1**_**R mRNA positive cells/nuclei in layers II (A – D) and V (E – H) of the human DLPFC.** CB_1_R silver grains (black dots) appear on small interneuron-like cells (large arrowheads) and larger pyramidal neuron-like cells (small arrows). Scale bar = 10 μm.

By *in situ* hybridisation we found a distinct laminar pattern of CB_1_R mRNA hybridisation signal in the grey matter, although scattered CB_1_R mRNA-positive signal was also found below the cortex in the white matter (small arrows, Figure 2). In the grey matter we found a dark band of increased CB_1_R mRNA immediately sub-adjacent to layer I, corresponding to layer II (large arrowheads, Figure 2). Another more diffuse band of increased CB_1_R mRNA signal was found in deeper cortical layers corresponding to layers V and VI, the width and intensity of which appeared to vary with age. There was no hybridisation detectable above background in sections incubated with the sense strand riboprobe (Additional file 2: Figure S2). Overall, *in situ* data indicated that CB_1_R mRNA expression decreased with age, particularly in the superficial cortical grey matter. Analysis of optical density of films generated from *in situ* hybridisation indicated that in all age groups, CB_1_R mRNA expression was highest in the supragranular cortical layer II (main effect: cortical layer F(5, 205) = 15.1, p < 0.001; simple contrast of layer II vs all other layers p < 0.001; Figure 3). Expression was highest in neonates and infants and lowest in adults, and these differences were statistically significant in layers II, III, V and VI (main effect: age group F(6, 41) = 2.54, p < 0.05; interaction effect: cortical layer x age F(30, 205) = 1.80, p < 0.01). Some layer-specific variation in the developmental pattern was evident. For example, in superficial layers II and III the school age group showed less pronounced reduction in CB_1_R mRNA density than in other layers; and in layer III only, teenagers showed significantly higher CB_1_R mRNA expression than both toddlers and adults. Silver grain clusters with a scatter diameter of 8–12 μm, representing the density of CB_1_R mRNA per cell, were visible 4–5 times above background levels on small interneuron-like cells in layers II, III, IV and VI (large arrowheads, Figure 4A – D). Clusters with a scatter diameter of 10 - 16 μm were visible on larger cells in layers III, V and VI (small arrows, Figure 4E - H).

### CB_1_R mRNA levels are not correlated with expression of the interneuron marker CCK

**Figure 5:**
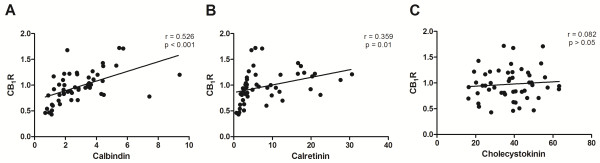
**Significant correlation of CB**_**1**_**R mRNA expression with mRNA for the interneuron markers calbindin (A) and calretinin (B), but not cholecystokinin (C)**.

Expression of CB_1_R mRNA, as measured by qPCR, was positively correlated with expression of mRNA for the interneuron markers calbindin (r = 0.526, p < 0.001) and calretinin (r = 0.359, p = 0.01; Figure 5A - B). There was no correlation between CB_1_R and CCK mRNA (r = 0.082, p > 0.05; Figure 5C).

2-AG synthesising enzyme mRNA (DAGL*α*) and hydrolysing enzyme mRNAs (MGL and ABHD6) show dynamic, divergent patterns across postnatal life.

**Figure 6:**
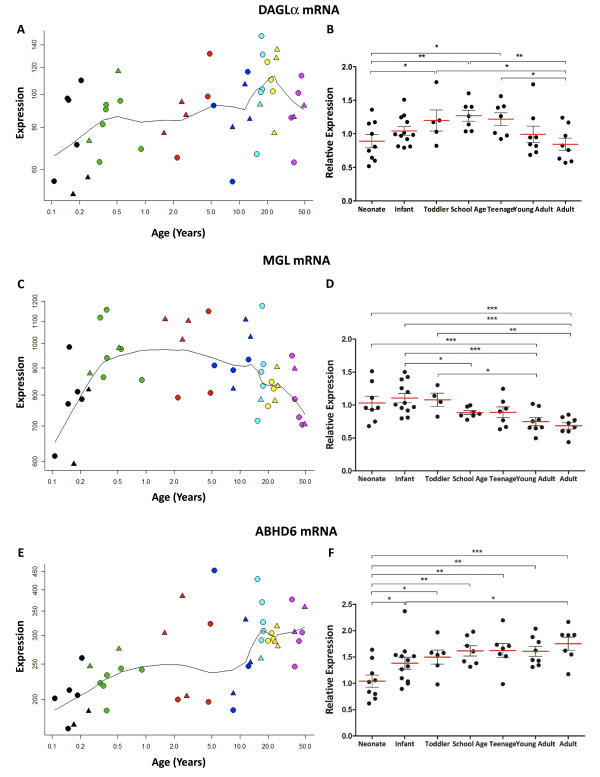
**DAGLα, MGL and ABHD6 mRNA in developing human DLPFC determined by microarray (A, C, E; y-axis, arbitrary units in log scale) across age (x- axis, log years), qPCR [B, D, F; y-axis, mean (+ S.E.M.) expression normalised to the geometric mean of four housekeeping genes] plotted by age group.** * P <0.05, ** P <0.01, *** P <0.001 (ANOVA/ANCOVA and post-hoc LSD).

Microarray analysis revealed a gradual increase in DAGLα expression (probeset 214128_at) from low neonate levels to a peak at young adulthood (r = 0.409, p < 0.01, Figure 6A). qPCR data indicated that DAGLα expression was significantly correlated with pH (r = 0.319, p < 0.05). However, due to variability and limited n, subsequent ANCOVA results showed that DAGLα expression approached, but did not reach a statistically significant change across development with our chosen primer and probes (F = 2.0, df = 6, 48, p = 0.08, Figure 6B). Based on microarray results, planned post-hoc LSD tests carried out on the qPCR expression data showed that compared with both neonates and adults, DAGLα mRNA expression was increased in toddlers (p < 0.05), school age (p < 0.01) and teenage individuals (p < 0.05), indicating a peak at school age.

MGL mRNA expression (probeset 211026_s_at) was quite variable in the microarray, showing an inverted U-shaped pattern across the human lifespan with a rapid increase until 6 months of age and then a steady decline starting after age 10 (quadratic regression p < 0.05, Figure 6C). qPCR analysis confirmed the developmental downregulation of MGL mRNA expression with age (ANOVA, F = 5.1, df = 6, 47, p < 0.001, Figure 6D), and post-hoc LSD tests showed that MGL expression was significantly reduced from infant levels by school age and continued to decrease from school age until adulthood. However, qPCR data failed to confirm the early increase in MGL mRNA from neonates to infants, suggesting that the prominent pattern was one of decline.

Microarray analysis showed that ABHD6 mRNA expression (probeset 45288_at) increased during human maturation by around 50% (r = 0.627, p < 0.001, Figure 6E). By qPCR, we saw a similar pattern, but we detected a steady increase in ABHD6 mRNA expression over postnatal development and maturation by qPCR, with a plateau from school age to young adulthood followed by an increase to adult levels [ANCOVA, F = 4.1, df = 6, 49, p = 0.002, Figure 6F; ABHD6 mRNA correlated with pH (r = 0.416, p = 0.001)]. Post-hoc LSD tests showed increased ABHD6 mRNA expression in all age groups compared with neonates, and in adults compared with infants (p < 0.05).

### Anandamide synthesising and hydrolysing enzyme mRNAs (NAPE-PLD and FAAH) increase across postnatal life

**Figure 7:**
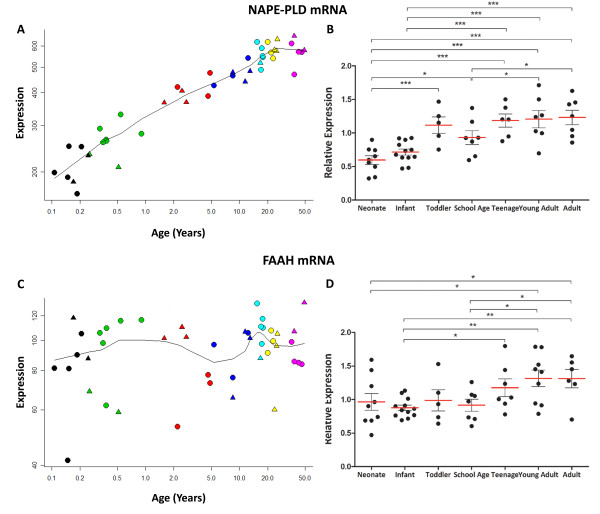
**NAPE-PLD and FAAH mRNA in developing human DLPFC determined by microarray (A, C; y-axis, arbitrary units in log scale) across age (x- axis, log years) and qPCR [B, D; y-axis, mean (+ S.E.M.) expression normalised to the geometric mean of four housekeeping genes] plotted by age group.** * P <0.05, ** P <0.01, *** P <0.001 (ANOVA/ANCOVA and post-hoc LSD).

Microarray analysis revealed a steady and large increase of almost 300% in NAPE-PLD mRNA expression (probeset 226041_at) from the neonate to adult groups (r = 0.959, p < 0.001, Figure 7A). By qPCR, we confirmed that NAPE-PLD mRNA expression increased steadily across development [ANCOVA, F = 6.8, df = 6, 45, p < 0.001, Figure 7B; correlated with PMI (r = −0.367, p < 0.001)]. Post-hoc LSD tests showed that compared with neonates NAPE-PLD mRNA expression was significantly increased in later life, from an 87% rise in toddlers to a 106% increase in adults.

FAAH mRNA expression (probeset 204231_s_at) was not altered across development in the microarray (r = 0.258, p = 0.09, Figure 7C). qPCR data indicated, however, that FAAH mRNA expression increased over the lifespan (ANOVA, F = 3.0, df = 6, 48, p = 0.02, Figure 7D), with post-hoc LSD tests showing that FAAH mRNA increased by 36% from neonates to young adulthood (p < 0.05).

## Discussion

**Figure 8:**
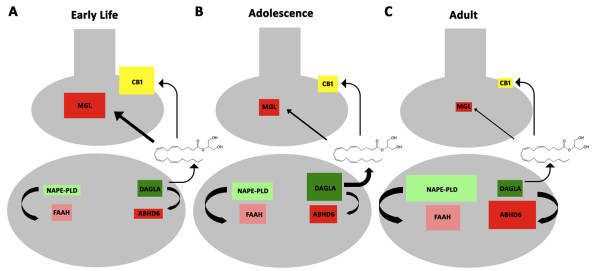
**Expression of mRNA for endocannabinoid receptor and metabolic enzymes across development.** qPCR data in our tissue cohort are represented schematically in the context of an inhibitory DLPFC synapse. Note that the size of each coloured mRNA reflects the relative magnitude of expression at different developmental points, not absolute mRNA levels. Arrow thickness represents relative prominence of signalling or enzymatic breakdown at each point. **A** Early life (neonate to toddler age) is characterised by robust potential for endocannabinoid modification of presynaptic neurotransmission (suggested by moderate 2-AG synthesis, low levels of postsynaptic 2-AG hydrolysis, high CB_1_R mRNA and low anandamide synthesis). **B** Adolescence is represented by increased potential for endocannabinoid modification of presynaptic input (suggested by elevated 2-AG synthesis), but reduced potency of such endocannabinoid modification compared with early life (reflected by reduced CB_1_R mRNA and increased anandamide synthesis). **C** By adulthood, modification of presynaptic neurotransmission by endocannabinoids may be minimal: despite reduced presynaptic 2-AG hydrolysis, there may be reduced capacity for retrograde 2-AG signalling due to reduced postsynaptic 2-AG synthesis and increased postsynaptic 2-AG hydrolysis, and CB_1_R mRNA is reduced. Anandamide turnover peaks at adulthood, suggesting increased prominence in signalling at CB_1_R or other receptors such as TRPV1.

Here, we present evidence for dynamic and non-concomitant changes in expression of mRNA for the CB_1_R and the metabolic enzymes for its major ligands during key epochs of human postnatal development (Figure 8). During early postnatal life (the period from neonate to toddlerhood), CB_1_R mRNA peaks in the DLPFC. Expression of the 2-AG synthetic (DAGLα) enzyme and the pre- and postsynaptic hydrolytic 2-AG enzymes MGL and ABHD6 begins to increase. Expression of one of the anandamide synthetic enzymes NAPE-PLD also increases steadily during early life while mRNA for FAAH, the anandamide hydrolytic enzyme, remains stable. At adolescence, CB_1_R mRNA is in decline compared with early life, particularly in cortical layer II. At this time, MGL has begun to decline while ABHD6 mRNA is increasing, compared with early postnatal life, suggesting an increased capacity for regulation of postsynaptic 2-AG release. The potential for anandamide turnover may increase in adolescence relative to earlier life given the rise in NAPE-PLD and, to a lesser extent, FAAH mRNA. By adulthood, CB_1_R mRNA is significantly reduced from early life levels, suggesting attenuation of control over synaptic neurotransmission mediated by this receptor, coincident with maturation of cognitive function. In contrast, the capacity for enzymatic control of anandamide turnover may continue to rise in adulthood, indicated by high mRNA expression of both NAPE-PLD and FAAH.

*In situ* hybridisation allowed us to determine the laminar distribution of CB_1_R mRNA in the DLPFC. CB_1_R mRNA is highest in cortical layer II in individuals aged from one month to 50 years. We observed silver grains indicative of CB_1_R mRNA expression densely clustered over small, interneuron-like cells in layers II, III, IV and VI and more sparsely collected over larger cells in layers III, V and VI. CB_1_Rs in primate DLPFC are primarily localised on CCK-positive inhibitory interneurons [16], but are also co-localised with the calcium binding proteins calbindin and calretinin in distinct subsets of cortical inhibitory interneurons [16,18-20]. We did not observe a correlation between CB_1_R and CCK mRNA, which increases steadily from neonatal age to adulthood [9]. However, CB_1_R mRNA expression was correlated with both calbindin, which follows an inverted U-shaped trajectory [9], and calretinin, which is highly expressed early in human development [9]. However, since CB_1_R [24], CCK [63,64] and calbindin mRNA [65] have been detected in pyramidal neurons, expression data may reflect heterogeneous origins of the mRNA signal, such as in layer V, which contains both pyramidal neurons and wide arbor interneurons. Further studies quantifying CB_1_R mRNA on specific cell types are necessary to resolve the developmental expression patterns of CB_1_R in specific interneuron types.

### Developmental changes in CB_1_R mRNA

Peak CB_1_R mRNA expression was observed at neonatal age by microarray but in toddlers by qPCR, a discrepancy that may arise from the differential preference for 3’ *versus* 5’ transcript detection between the two techniques. Laminar analysis of *in situ* hybridisation indicated that CB_1_R mRNA expression peaks in neonates and infants in most cortical layers. These findings are confirmed by recent data from a different study showing a similar trajectory from early life to adulthood, with CB_1_R mRNA peaking at neonatal age then decreasing until adulthood [66].

The robust decrease in CB_1_R mRNA expression in layer II from early life to adulthood in our human cohort agrees with previous findings in primate [67] and suggests strong regulation of neurotransmission by CB_1_Rs early in life (Figure 8). It is tempting to speculate that this CB_1_R down-regulation results in a gradual reduction in CB_1_R-mediated suppression of inhibitory presynaptic neurotransmission. Indeed, the first putative drop in CB_1_R mRNA expression in layer II occurs around the toddler/school age group, which represents a period of increased inhibitory control in the prefrontal cortex marked by dynamic changes in inhibitory interneuron marker expression [9] and increased working memory ability [68].

### Endocannabinoids in early postnatal life

The shape of the postnatal trajectory of DAGLα mRNA suggests that the capacity for synthesis of 2-AG, the major cortical endocannabinoid, increases during early postnatal life. In early life, 2-AG synthesis is also catalysed by the beta isoform of DAGL, but since expression of this isoform may decrease very early in postnatal life [38] it was not measured in the present study. 2-AG tone is principally regulated by the presynaptic hydrolytic enzyme MGL [44], which showed reduced mRNA expression over postnatal life after peaking in infancy, and also by the postsynaptic enzyme ABHD6, expression of which increased steadily from neonatal age to adulthood. In early development, MGL co-localises with CB_1_Rs in presynaptic terminals of developing cortical pyramidal neurons in order to regulate corticofugal axon fasciculation [50]. Thus the role of MGL in controlling 2-AG availability may diminish once appropriate cortical-origin connections are in place. Our results suggest that the major endogenous ligand acting on the CB_1_R, 2-AG, may be somewhat unstable early in human life due to quite high variability in mRNA controlling synthesis and breakdown within individuals less than 5 years of age. We suggest that 2-AG may be especially influential and subject to increased regulation in early postnatal life when the CB_1_R and presynaptic breakdown enzyme for 2-AG are at developmentally high levels.

### Endocannabinoids in adolescence

By adolescence there is a significant decrease in CB_1_R expression, suggesting an overall decrease in sensitivity to the effects of released endocannabinoids on neurotransmitter release. At this age, working memory performance reaches near adult levels with concomitant increases in DLPFC activity recruitment [4,5]. However, at adolescence, DAGLα has not started to decline and MGL mRNA has just begun to decrease, while ABHD6 mRNA continues to increase, suggesting that capacity for 2-AG hydrolysis in pre- and postsynaptic compartments may be anatomically balanced at this age. Thus, maintaining the earlier steady-state levels of 2-AG, while incorporating more temporal/spatial control of 2-AG action in the face of CB_1_R down-regulation, may be important during adolescence. This is an intriguing possibility and agrees with the increasing endocannabinoid-mediated suppression of neurotransmitter release observed in postnatal rat hippocampus until early adolescence [52]. Taken together, our data imply that the capacity for 2-AG signalling remains fairly steady between early postnatal life and adolescence then may decline. The gradual increase in expression at adolescence of the mRNA for anandamide hydrolytic enzyme FAAH, which is localised postsynaptically [40], suggests that at this point there is increased potential for control over the timing of anandamide availability for signalling at CB_1_R as well.

### Endocannabinoids in adulthood

CB_1_R mRNA continues to decline until young adulthood, when individuals have attained mature levels of inhibitory control over cortical excitability. Combined with the early life increase in DAGLα and its decline in adulthood (compared with toddlerhood, school age and adolescence), our data is broadly consistent with previous work in the rat indicating that 2-AG is higher earlier in postnatal life and declines with maturation [69]. As maturation progresses the decline in DAGLα and MGL and increase in ABHD6 mRNA reflect the potential for reduction in the amount of 2-AG available for release from the postsynaptic terminal as compared with early postnatal life (Figure 8). Combined with reduced CB_1_R expression at the same age, reduced 2-AG availability after adolescence might ‘release’ the endocannabinoid-mediated suppression of presynaptic neurotransmission to allow greater inhibitory input to cortical circuitry. Our data suggest an overall increase in the capacity for anandamide synthesis via the NAPE-PLD pathway during development and maturation of the human DLPFC. Neonatal NAPE-PLD expression levels increase threefold by adulthood, extending to humans previous findings of increased cortical NAPE-PLD mRNA expression from postnatal day 14 to 56 in rats [70] and suggesting that, as in rat brain, anandamide synthesis may be higher in maturity than in early life [69]. There are several possible functional implications of these observations. Anandamide induces long-term depression of inhibitory synapses via transient receptor potential vanilloid 1 (TRPV1) receptors in hippocampus, striatum and developing midbrain [71-73], and since TRPV1 receptors are expressed cortically [74] it is possible that anandamide facilitates excitatory neurotransmission in this region in a similar manner to its effects in the hippocampus. However, if this were the case, then increased anandamide signalling would be expected to result in decreased, rather than increased inhibitory tone as maturation proceeds. On the other hand, anandamide opposes striatal 2-AG signalling by down-regulating 2-AG production [75], suggesting the possibility that increased NAPE-PLD expression results in greater synthetic capacity for anandamide production and consequent down-regulation of 2-AG production towards adulthood. Finally, our data must be interpreted carefully in light of the other enzymatic pathways for anandamide synthesis, including GDE1, ABHD4 and PTPN22. Future studies will benefit from investigating the postnatal developmental expression of these mRNAs.

## Conclusions

Overall, our data show that human early postnatal life, adolescence and adulthood are characterised by distinct differences in capacities for endocannabinoid synthesis, breakdown and receptor expression, which we summarise in Figure 8. While the varying circumstances surrounding death and variable post-mortem intervals are limitations of using human brain material, these factors did not appear to obscure the major impact of age on endocannabinoid system mRNAs nor did they have a major impact on the measurements in our study. Our findings may help to identify pathophysiological mechanisms in developmental disorders characterised by compromised cortical inhibitory function such as schizophrenia [9,11,76,77] and autism [78]. For example, disruption of this dynamic postnatal maturation by exogenous cannabinoid exposure might impact processes such as cortical network oscillations, which are governed by inhibitory interneuron activity and coordinate behavioural processes such as discrimination during decision-making [1] and have been reported to be susceptible to suppression by CB_1_R agonists [79,80]. Importantly, since adolescence is the most likely period of commencement of cannabis use, our findings emphasise the need to determine how exposure to exogenous cannabis constituents such as THC during this period affects the changes in the endocannabinoid system and its role as a gatekeeper of neurotransmission.

## Methods

### Subjects

Tissue from the middle frontal gyrus of 69 cases aged 39 days to 49 years was obtained from the University of Maryland Brain and Tissue Bank for Developmental Disorders (NICHHD contract no. NO1 = HD8-3283) following informed consent from next of kin. This study was carried out in accordance with the latest version of the Declaration of Helsinki after review by the University of NSW Human Research Ethics Committee (HREC #07261). Cases were grouped and labelled according to age, and demographic details of this cohort (Table 1) have been described previously [81].

**Table 1:** Summary of developmental cohort used for experiments

**Group**	**Age (years)**	**Gender**	**pH**	**PMI**	**RIN**	**n**
*Microarray*						
Neonate	0.11 - 0.24	2F, 5M	6.60 ± 0.05	22.71 ± 2.40	7.27 ± 0.18	7
Infant	0.25 - 0.91	2F, 6M	6.69 ± 0.05	17.63 ± 2.72	7.57 ± 0.17	8
Toddler	1.58 - 4.86	3F, 3M	6.74 ± 0.07	25.67 ± 3.90	6.91 ± 0.24	6
School Age	7.84 - 12.97	3F, 3M	6.73 ± 0.06	14.67 ± 2.22	7.03 ± 0.31	6
Teenage	16.34 - 17.82	1F, 4M	6.75 ± 0.05	18.40 ± 2.16	6.26 ± 0.53	5
Young Adult	20.14 - 25.38	2F, 4M	6.75 ± 0.07	12.00 ± 2.21	6.91 ± 0.24	6
Adult	35.99 - 49.22	2F, 4M	6.68 ± 0.09	13.83 ± 2.18	6.78 ± 0.25	6
*qPCR*						
Neonate	0.11 - 0.24	4F, 5M	6.56 ± 0.05	22.11 ± 1.88	7.01 ± 0.27	9
Infant	0.25 - 0.91	5F, 8M	6.61 ± 0.05	17.46 ± 1.76	7.19 ± 0.19	13
Toddler	1.58 - 4.86	4F, 4M	6.77 ± 0.06	19.25 ± 1.89	6.85 ± 0.25	8
School Age	7.84 - 12.97	4F, 3M	6.69 ± 0.07	14.43 ± 1.95	6.88 ± 0.24	7
Teenage	15 - 17.69	2F, 5M	6.74 ± 0.03	17.86 ± 1.47	6.60 ± 0.28	7
Young Adult	20.14 - 25.38	3F, 6M	6.67 ± 0.08	13.67 ± 2.75	6.73 ± 0.22	9
Adult	35.99 - 49.22	3F, 5M	6.60 ± 0.10	13.38 ±1 .63	6.53 ± 0.27	8
*In situ hybridisation*						
Neonate	0.11 - 0.24	4F, 5M	6.60 ± 0.05	22.11 ± 1.88	7.01 ± 0.27	9
Infant	0.25 - 0.91	5F, 8M	6.61 ± 0.05	17.46 ± 1.76	7.20 ± 0.19	13
Toddler	1.58 - 4.86	5F, 4M	6.74 ± 0.06	22.00 ± 3.22	6.83 ± 0.22	9
School Age	7.84 - 12.97	4F, 3M	6.69 ± 0.07	14.43 ± 1.95	6.88 ± 0.24	7
Teenage	15 - 17.69	2F, 4M	6.75 ± 0.04	16.67 ± 1.02	6.64 ± 0.33	6
Young Adult	21.93 - 25.38	3F, 4M	6.67 ± 0.09	14.43 ± 3.20	6.83 ± 0.25	7
Adult	35.99 – 48.69	1F, 4M	6.45 ± 0.10	12.60 ± 1.60	6.39 ± 0.35	5

### Total RNA isolation and RNA quality assessment

RNA isolation, quality assessment, microarray and qPCR analyses were conducted following established protocols [82]. Total RNA was extracted for microarray and qPCR analysis from 300 mg gray matter using Trizol (Invitrogen, Carlsbad, California) according to the manufacturer’s instructions [83]. The quality of total RNA was determined using the Agilent Bioanalyzer 2100 (Agilent Technologies, Palo Alto, California). 100-200 ng RNA was applied to an RNA 6000 Nano LabChip, without heating prior to loading. The RNA integrity number (RIN) was used as an indicator of RNA quality, ranging from 1 – 10 (lowest - highest quality). Six samples were excluded with RIN <5.2. Cases did not differ significantly within each age group according to brain pH or RIN.

### Microarray experimental design

Total RNA (n = 45) was purified through Qiagen RNA miniKit columns (Qiagen Inc, Valencia, California) according to the manufacturer’s protocol. Purified total RNA was then prepared according to the Affymetrix protocol http://www.affymetrix.com[84]] and hybridised to HG-U133 version 2.0+ (GeneChips, Affymetrix California). Hybridised arrays were subjected to rigorous quality control including analysis of 5’:3’ ratios (included range 0.40-0.79), percent present (included range 37 - 47%), average pair-wise correlation analysis and principal component analysis, resulting in the exclusion of 3 individuals. Affymetrix Microarray Suite (MAS 5.0) and the Bioconductor package were used for image processing and data normalisation respectively. Probe sets that were 50% present in at least one of the age subgroups were retained in the analysis (33,210 probe sets retained; 61% of total number). Microarray data are available in the Gene Expression Omnibus archive under series accession number GSE13564.

### Microarray and qPCR analyses

Results from the microarray experiment were confirmed using qPCR (n = 61). cDNA was synthesised in 3 reactions of 3 μg of total RNA using the Superscript First-Strand Synthesis Kit (Invitrogen) according to the manufacturer’s protocol. Pre-designed TaqMan Gene Expression Assays (Applied Biosystems, Foster City, California) were chosen for the CB_1_R (CNR1; Hs00275634_m1), the 2-AG synthesising enzyme diacylglycerol lipase alpha (DAGLα, Hs00391374_m1), the 2-AG inactivating enzymes monoglyceride lipase (MGL, Hs00200752_m1) and abhydrolase domain containing 6 (ABHD6, Hs0097789_m1), the anandamide synthesizing enzyme N-acylphosphatidylethanolamine phospholipase D (NAPE-PLD, Hs00419593_m1) and the anandamide inactivating enzyme fatty acid amide hydrolase (FAAH, Hs00155015_m1). qPCR was performed with an ABI Prism 7900HT Fast real-time PCR system with a 384-well format. Measurements were performed in triplicate and relative quantities determined from a seven-point standard curve. Control wells containing no cDNA template displayed no amplification. Efficiencies of the qPCR reactions ranged from 80% to 100%, and r^2^ values were between 0.97 and 1.00. Outliers were excluded from qPCR analysis if their normalised expression values were greater than 2 standard deviations from the group mean. Expression levels were normalised to the geometric mean of four ‘housekeeper’ genes that did not change expression with development: hydroxymethylbilane synthase (HMBS, Hs00609297_m1), glucuronidase-b (GUSB, Hs99999908_m1), ubiquitin C (UBC, Hs00824723_m1) and cyclophilin A (PPIA, Hs99999904_m1) [81].

### Riboprobe design

A 247 bp fragment of the human CNR1 gene cDNA, corresponding to bps 937–1183, was inserted into a pCRII vector (Invitrogen, CA, USA). Antisense riboprobes and sense strand RNAs were generated from linearised plasmid using SP6 and T7 polymerases respectively and an *in vitro* transcription kit as recommended by the manufacturer (Promega, WI, USA). [^35^S] antisense and sense riboprobes were labelled to a specific activity of ~2 x 10^9^ cpm/μg by addition of radiolabelled UTP and were purified by ethanol precipitation.

### In situ *hybridisation*

14-μm coronal sections of the middle frontal gyrus from the developmental cohort tissue were used (n = 56). *In situ* hybridisation histochemistry was performed as previously described [85], using two slides per case. After hybridisation and washing steps, sections were exposed to autoradiographic film (Bio-Max, Kodak) for 3 weeks. Sections were then dipped in photographic emulsion (Kodak, type II NTB) for 13 weeks and then developed and counterstained with thionine. All sections were assayed together to limit interassay variability.

### Image analysis

Calibrated densitometric analysis (ImageJ, National Institutes of Health, http://rsb.info.nih.gov/ij/) was conducted, blind to age, on Brodmann’s area 46 of the DLPFC delineated microscopically from adjacent Nissl-stained sections. For each section, three randomly placed lines (415 μm width) were drawn perpendicular to the pial surface, traversing the entire cortical grey matter. The density of CB_1_R mRNA as a function of cortical depth was computed from continuous optical density measurements recorded along the length of these lines, using radioactive standards for comparison (American Radiolabeled Chemicals Inc., MO, USA). The percentage of full cortical width corresponding to the individual lamina was determined from published guidelines as follows: I (2-6%), II (14-18%), III (20-42%), IV (46-50%), V (54-68%) and VI (72-92%) [86,87].

### Statistical analysis

Normalisation and analysis of microarray data were performed using R (http://www.r-project.org) and Bioconductor software (http://www.bioconductor.org), with which differential gene expression across chronological age was analysed using linear regression. The R program was used to draw regression lines of best fit depending on the profile of gene expression changes with age. qPCR and *in situ* hybridisation data were analysed using PASW Statistics 18 (SPSS Inc., IL, USA). Differences in normalised mRNA expression detected by qPCR were tested with one-way analysis of variance (ANOVA) with age group as the grouping variable in all analyses. Pearson’s correlation analyses were performed on normalised qPCR mRNA expression and demographic variables, and analysis of covariance (ANCOVA) was conducted where significant correlations were observed with pH, post-mortem interval (PMI) and RIN as continuous variables. Differences in optical density of CB_1_R mRNA detected by *in situ* hybridisation were tested with two-way repeated measures ANOVA with age group as the between-subjects variable and cortical layer as the within-subjects variable. Fisher’s least significant difference (LSD) post-hoc analyses were used to identify significant differences between age groups. Simple linear regression was performed to identify predictive relationships between normalised expression of CB_1_R mRNA detected by qPCR and interneuron marker mRNAs of interest that were previously measured in the same cohort [9].

## Competing interests

The author declared that they have no competing interest.

## Authors’ contributions

CSW and LL contributed the design, execution, data analysis and interpretation of the study, JL conducted the in situ data analysis, and MJW provided neuroanatomical expertise, access to the tissue and provided comments on the manuscript. All authors read and approved the final manuscript.

## Funding

This work was supported by the Schizophrenia Research Institute, utilising infrastructure funding from New South Wales Health and the Macquarie Group Foundation, Neuroscience Research Australia and the University of New South Wales.

## Supplementary Material

Additional file 1 **Figure S1.** Geometric mean of expression of HMBS, GUSB, UBC and PPIA mRNA in human DLPFC determined by qPCR [y-axis, mean (+ S.E.M.)] expression plotted by age group. n = 7 – 13. Click here for file

Additional file 2 **Figure S2.** Representative autoradiograms from sections from two school-aged individuals showing A CB_1_R mRNA hybridisation after antisense strand riboprobe incubation and Bnegligible CB_1_R mRNA hybridisation after sense strand riboprobe incubation. Click here for file

## References

[B1] McBain CJ Fisahn A Interneurons unbound Nat Rev Neurosci 2001 2 1 11 23 11253355 10.1038/35049047

[B2] Markram H Toledo-Rodriguez M Wang Y Gupta A Silberberg G Wu C Interneurons of the neocortical inhibitory system Nat Rev Neurosci 2004 5 10 793 807 10.1038/nrn1519 15378039

[B3] Diamond A Goldman-Rakic PS Comparison of human infants and rhesus monkeys on Piaget's AB task: evidence for dependence on dorsolateral prefrontal cortex Exp Brain Res 1989 74 1 24 40 2924839 10.1007/BF00248277

[B4] Crone EA Wendelken C Donohue S van Leijenhorst L Bunge SA Neurocognitive development of the ability to manipulate information in working memory Proc Natl Acad Sci U S A 2006 103 24 9315 9320 10.1073/pnas.0510088103 16738055 PMC1472660

[B5] Luna B Garver KE Urban TA Lazar NA Sweeney JA Maturation of cognitive processes from late childhood to adulthood Child Dev 2004 75 5 1357 1372 10.1111/j.1467-8624.2004.00745.x 15369519

[B6] Bourgeois J-P Goldman-Rakic PS Rakic P Synaptogenesis in the prefrontal cortex of rhesus monkeys Cereb Cortex 1994 4 1 78 96 10.1093/cercor/4.1.78 8180493

[B7] Fung SJ Webster MJ Weicker CS Expression of VGluT1 and VGAT mRNAs in human dorsolateral prefrontal cortex during development and in schizophrenia Brain Res 2011 1388 22 31 21396926 10.1016/j.brainres.2011.03.004

[B8] Webster MJ Elashoff M Weickert CS Molecular evidence that cortical synaptic growth predominates during the first decade of life in humans Int J Dev Neurosci 2011 29 3 225 236 10.1016/j.ijdevneu.2010.09.006 20888897

[B9] Fung SJ Webster MJ Sivagnanasundaram S Duncan C Elashoff M Weickert CS Expression of interneuron markers in the dorsolateral prefrontal cortex of the developing human and in schizophrenia Am J Psychiatry 2010 167 12 1479 1488 10.1176/appi.ajp.2010.09060784 21041246

[B10] Fillman SG Duncan CE Webster MJ Elashoff M Weickert CS Developmental co-regulation of the [beta] and [gamma] GABAA receptor subunits with distinct [alpha] subunits in the human dorsolateral prefrontal cortex Int J Dev Neurosci 2010 28 6 513 519 10.1016/j.ijdevneu.2010.05.004 20609421

[B11] Duncan CE Webster MJ Rothmond DA Bahn S Elashoff M Shannon Weickert C Prefrontal GABAA receptor [alpha]-subunit expression in normal postnatal human development and schizophrenia J Psychiatr Res 2010 44 10 673 681 10.1016/j.jpsychires.2009.12.007 20100621

[B12] Hashimoto T Nguyen QL Rotaru D Keenan T Arion D Beneyto M Gonzalez-Burgos G Lewis DA Protracted developmental trajectories of GABAA receptor alpha1 and alpha2 subunit expression in primate prefrontal cortex Biol Psychiatry 2009 65 12 1015 1023 10.1016/j.biopsych.2009.01.004 19249749 PMC2882199

[B13] Ben-Ari Y Gaiarsa J-L Tyzio R Khazipov R GABA: a pioneer transmitter that excites immature neurons and generates primitive oscillations Physiol Rev 2007 87 4 1215 1284 10.1152/physrev.00017.2006 17928584

[B14] Rivera C Voipio J Payne JA Ruusuvuori E Lahtinen H Lamsa K Pirvola U Saarma M Kaila K The K+/Cl- co-transporter KCC2 renders GABA hyperpolarizing during neuronal maturation Nature 1999 397 6716 251 255 10.1038/16697 9930699

[B15] Hyde TM Lipska BK Ali T Mathew SV Law AJ Metitiri OE Straub RE Ye T Colantuoni C Herman MM Expression of GABA signaling molecules KCC2, NKCC1, and GAD1 in cortical development and schizophrenia J Neurosci 2011 31 30 11088 11095 10.1523/JNEUROSCI.1234-11.2011 21795557 PMC3758549

[B16] Eggan SM Melchitzky DS Sesack SR Fish KN Lewis DA Relationship of cannabinoid CB1 receptor and cholecystokinin immunoreactivity in monkey dorsolateral prefrontal cortex Neuroscience 2010 169 4 1651 1661 10.1016/j.neuroscience.2010.06.011 20542094 PMC3030191

[B17] Katona I Sperlagh B Sik A Kafalvi A Vizi ES Mackie K Freund TF Presynaptically located CB1 cannabinoid receptors regulate GABA release from axon terminals of specific hippocampal interneurons J Neurosci 1999 19 11 4544 4558 10341254 10.1523/JNEUROSCI.19-11-04544.1999 PMC6782612

[B18] Morozov YM Torii M Rakic P Origin, early commitment, migratory routes, and destination of cannabinoid type 1 receptor-containing interneurons Cereb Cortex 2009 19 suppl_1 i78 i89 19346272 10.1093/cercor/bhp028 PMC3584650

[B19] Marsicano G Lutz B Expression of the cannabinoid receptor CB1 in distinct neuronal subpopulations in the adult mouse forebrain Eur J Neurosci 1999 11 12 4213 4225 10.1046/j.1460-9568.1999.00847.x 10594647

[B20] Bodor AL Katona I Nyiri G Mackie K Ledent C Hajos N Freund TF Endocannabinoid signaling in rat somatosensory cortex: laminar differences and involvement of specific interneuron types J Neurosci 2005 25 29 6845 6856 10.1523/JNEUROSCI.0442-05.2005 16033894 PMC6725346

[B21] Cristino L de Petrocellis L Pryce G Baker D Guglielmotti V Di Marzo V Immunohistochemical localization of cannabinoid type 1 and vanilloid transient receptor potential vanilloid type 1 receptors in the mouse brain Neuroscience 2006 139 4 1405 1415 10.1016/j.neuroscience.2006.02.074 16603318

[B22] Monory K Massa F Egertova M Eder M Blaudzun H Westenbroek R Kelsch W Jacob W Marsch R Ekker M The endocannabinoid system controls key epileptogenic circuits in the hippocampus Neuron 2006 51 4 455 466 10.1016/j.neuron.2006.07.006 16908411 PMC1769341

[B23] Kawamura Y Fukaya M Maejima T Yoshida T Miura E Watanabe M Ohno-Shosaku T Kano M The CB1 cannabinoid receptor is the major cannabinoid receptor at excitatory presynaptic sites in the hippocampus and cerebellum J Neurosci 2006 26 11 2991 3001 10.1523/JNEUROSCI.4872-05.2006 16540577 PMC6673964

[B24] Freund TF Katona I Piomelli D Role of endogenous cannabinoids in synaptic signaling Physiol Rev 2003 83 3 1017 1066 12843414 10.1152/physrev.00004.2003

[B25] Pertwee RG Howlett AC Abood ME Alexander SPH Di Marzo V Elphick MR Greasley PJ Hansen HS Kunos G Mackie K International Union of Basic and Clinical Pharmacology. LXXIX. Cannabinoid Receptors and Their Ligands: Beyond CB1 and CB2 Pharmacol Rev 2010 62 4 588 631 10.1124/pr.110.003004 21079038 PMC2993256

[B26] Trettel J Levine ES Endocannabinoids mediate rapid retrograde signaling at interneuron right-arrow pyramidal neuron synapses of the neocortex J Neurophysiol 2003 89 4 2334 2338 12686587 10.1152/jn.01037.2002

[B27] Fortin DA Levine ES Differential effects of endocannabinoids on glutamatergic and GABAergic inputs to layer 5 pyramidal neurons Cereb Cortex 2007 17 1 163 174 16467564 10.1093/cercor/bhj133

[B28] Wilson RI Nicoll RA Endogenous cannabinoids mediate retrograde signalling at hippocampal synapses Nature 2001 410 6828 588 592 10.1038/35069076 11279497

[B29] Ade KK Lovinger DM Anandamide regulates postnatal development of long-term synaptic plasticity in the rat dorsolateral striatum J Neurosci 2007 27 9 2403 2409 10.1523/JNEUROSCI.2916-06.2007 17329438 PMC6673491

[B30] Azad SC Monory K Marsicano G Cravatt BF Lutz B Zieglgänsberger W Rammes G Circuitry for associative plasticity in the amygdala Involves endocannabinoid signaling J Neurosci 2004 24 44 9953 9961 10.1523/JNEUROSCI.2134-04.2004 15525780 PMC6730232

[B31] Lafourcade M Elezgarai I Mato S Bakiri Y Grandes P Manzoni OJ Molecular components and functions of the endocannabinoid system in mouse prefrontal cortex PLoS One 2007 2 8 e709 10.1371/journal.pone.0000709 17684555 PMC1933592

[B32] Iremonger KJ Kuzmiski JB Baimoukhametova DV Bains JS Dual Regulation of Anterograde and Retrograde Transmission by Endocannabinoids J Neurosci 2011 31 33 12011 12020 10.1523/JNEUROSCI.2925-11.2011 21849561 PMC6623194

[B33] Okamoto Y Morishita J Tsuboi K Tonai T Ueda N Molecular characterization of a phospholipase D generating anandamide and its congeners J Biol Chem 2004 279 7 5298 5305 14634025 10.1074/jbc.M306642200

[B34] Ueda N Okamoto Y Morishita J N-acylphosphatidylethanolamine-hydrolyzing phospholipase D: A novel enzyme of the [beta]-lactamase fold family releasing anandamide and other N-acylethanolamines Life Sci 2005 77 14 1750 1758 10.1016/j.lfs.2005.05.018 15949819

[B35] Simon GM Cravatt BF Anandamide biosynthesis catalyzed by the phosphodiesterase GDE1 and detection of glycerophospho-N-acyl ethanolamine precursors in mouse brain J Biol Chem 2008 283 14 9341 9349 10.1074/jbc.M707807200 18227059 PMC2431036

[B36] Liu J Wang L Harvey-White J Huang BX Kim H-Y Luquet S Palmiter RD Krystal G Rai R Mahadevan A Multiple pathways involved in the biosynthesis of anandamide Neuropharmacology 2008 54 1 1 7 10.1016/j.neuropharm.2007.05.020 17631919 PMC2219543

[B37] Liu J Wang L Harvey-White J Osei-Hyiaman D Razdan R Gong Q Chan AC Zhou Z Huang BX Kim H-Y A biosynthetic pathway for anandamide Proc Natl Acad Sci U S A 2006 103 36 13345 13350 10.1073/pnas.0601832103 16938887 PMC1557387

[B38] Bisogno T Howell F Williams G Minassi A Cascio MG Ligresti A Matias I Schiano-Moriello A Paul P Williams E-J Cloning of the first sn1-DAG lipases points to the spatial and temporal regulation of endocannabinoid signaling in the brain J Cell Biol 2003 163 3 463 468 10.1083/jcb.200305129 14610053 PMC2173631

[B39] Cravatt BF Lichtman AH The enzymatic inactivation of the fatty acid amide class of signaling lipids Chem Phys Lipids 2002 121 1–2 135 148 12505696 10.1016/s0009-3084(02)00147-0

[B40] Egertova M Cravatt BF Elphick MR Comparative analysis of fatty acid amide hydrolase and CB(1) cannabinoid receptor expression in the mouse brain: evidence of a widespread role for fatty acid amide hydrolase in regulation of endocannabinoid signaling Neuroscience 2003 119 2 481 496 10.1016/S0306-4522(03)00145-3 12770562

[B41] Saario SM Savinainen JR Laitinen JT Jarvinen T Niemi R Monoglyceride lipase-like enzymatic activity is responsible for hydrolysis of 2-arachidonoylglycerol in rat cerebellar membranes Biochem Pharmacol 2004 67 7 1381 1387 10.1016/j.bcp.2003.12.003 15013854

[B42] Blankman JL Simon GM Cravatt BF A comprehensive profile of brain enzymes that hydrolyze the endocannabinoid 2-arachidonoylglycerol Chem Biol 2007 14 12 1347 1356 10.1016/j.chembiol.2007.11.006 18096503 PMC2692834

[B43] Marrs WR Blankman JL Horne EA Thomazeau A Lin YH Coy J Bodor AL Muccioli GG Hu SS-J Woodruff G The serine hydrolase ABHD6 controls the accumulation and efficacy of 2-AG at cannabinoid receptors Nat Neurosci 2010 13 8 951 957 10.1038/nn.2601 20657592 PMC2970523

[B44] Schlosburg JE Blankman JL Long JZ Nomura DK Pan B Kinsey SG Nguyen PT Ramesh D Booker L Burston JJ Chronic monoacylglycerol lipase blockade causes functional antagonism of the endocannabinoid system Nat Neurosci 2010 13 9 1113 1119 10.1038/nn.2616 20729846 PMC2928870

[B45] Dinh TP Carpenter D Leslie FM Freund TF Katona I Sensi SL Kathuria S Piomelli D Brain monoglyceride lipase participating in endocannabinoid inactivation Proc Natl Acad Sci U S A 2002 99 16 10819 10824 10.1073/pnas.152334899 12136125 PMC125056

[B46] Oudin MJ Gajendra S Williams G Hobbs C Lalli G Doherty P Endocannabinoids regulate the migration of subventricular zone-derived neuroblasts in the postnatal brain J Neurosci 2011 31 11 4000 4011 10.1523/JNEUROSCI.5483-10.2011 21411643 PMC6623539

[B47] Mulder J Aguado T Keimpema E Barabas K Ballester Rosado CJ Nguyen L Monory K Marsicano G Di Marzo V Hurd YL Endocannabinoid signaling controls pyramidal cell specification and long-range axon patterning Proc Natl Acad Sci U S A 2008 0803545105 10.1073/pnas.0803545105 PMC2438381 18562289

[B48] Berghuis P Dobszay MB Wang X Spano S Ledda F Sousa KM Schulte G Ernfors P Mackie K Paratcha G Endocannabinoids regulate interneuron migration and morphogenesis by transactivating the TrkB receptor Proc Natl Acad Sci U S A 2005 102 52 19115 19120 10.1073/pnas.0509494102 16357196 PMC1323195

[B49] Berghuis P Rajnicek AM Morozov YM Ross RA Mulder J Urban GM Monory K Marsicano G Matteoli M Canty A Hardwiring the brain: endocannabinoids shape neuronal connectivity Science 2007 316 5828 1212 1216 10.1126/science.1137406 17525344

[B50] Keimpema E Barabas K Morozov YM Tortoriello G Torii M Cameron G Yanagawa Y Watanabe M Mackie K Harkany T Differential subcellular recruitment of monoacylglycerol lipase generates spatial specificity of 2-arachidonoyl glycerol signaling during axonal pathfinding J Neurosci 2010 30 42 13992 14007 10.1523/JNEUROSCI.2126-10.2010 20962221 PMC2987617

[B51] Wu CS Zhu J Wager-Miller J Wang S O'Leary D Monory K Lutz B Mackie K Lu HC Requirement of cannabinoid CB(1) receptors in cortical pyramidal neurons for appropriate development of corticothalamic and thalamocortical projections Eur J Neurosci 2010 32 5 693 706 10.1111/j.1460-9568.2010.07337.x 21050275 PMC2970673

[B52] Zhu PJ Lovinger DM Developmental alteration of endocannabinoid retrograde signaling in the hippocampus J Neurophysiol 2010 103 2 1123 1129 10.1152/jn.00327.2009 20007500 PMC2822685

[B53] Malone DT Hill MN Rubino T Adolescent cannabis use and psychosis: epidemiology and neurodevelopmental models Br J Pharmacol 2010 160 3 511 522 10.1111/j.1476-5381.2010.00721.x 20590561 PMC2931552

[B54] Verrico CD Liu S Bitler EJ Gu H Sampson AR Bradberry CW Lewis DA Delay- and dose-dependent effects of [delta]9-tetrahydrocannabinol administration on spatial and object working memory tasks in adolescent rhesus monkeys Neuropsychopharmacology 2012 37 6 1357 1366 10.1038/npp.2011.321 22218091 PMC3327841

[B55] Dragt S Nieman DH Becker HE van de Fliert R Dingemans PM de Haan L van Amelsvoort TA Linszen DH Age of onset of cannabis use is associated with age of onset of high-risk symptoms for psychosis Can J Psychiatry 2010 55 3 165 20370967 10.1177/070674371005500308

[B56] Cohen M Rasser PE Peck G Carr VJ Ward PB Thompson PM Johnston P Baker A Schall U Cerebellar grey-matter deficits, cannabis use and first-episode schizophrenia in adolescents and young adults Int J Neuropsychopharmacol 2011 FirstView 1 11 21557880 10.1017/S146114571100068X

[B57] Arseneault L Cannon M Poulton R Murray R Caspi A Moffitt TE Cannabis use in adolescence and risk for adult psychosis: longitudinal prospective study BMJ 2002 325 7374 1212 1213 10.1136/bmj.325.7374.1212 12446537 PMC135493

[B58] Ehrenreich H Rinn T Kunert HJ Moeller MR Poser W Schilling L Gigerenzer G Hoehe MR Specific attentional dysfunction in adults following early start of cannabis use Psychopharmacology 1999 142 3 295 301 10.1007/s002130050892 10208322

[B59] Mato S Del Olmo E Pazos A Ontogenetic development of cannabinoid receptor expression and signal transduction functionality in the human brain Eur J Neurosci 2003 17 9 1747 1754 10.1046/j.1460-9568.2003.02599.x 12752773

[B60] Glass M Dragunow M Faull RL Cannabinoid receptors in the human brain: a detailed anatomical and quantitative autoradiographic study in the fetal, neonatal and adult human brain Neuroscience 1997 77 2 299 318 10.1016/S0306-4522(96)00428-9 9472392

[B61] Biegon A Kerman IA Autoradiographic study of pre- and postnatal distribution of cannabinoid receptors in human brain Neuroimage 2001 14 6 1463 1468 10.1006/nimg.2001.0939 11707102

[B62] Wang X Dow-Edwards D Keller E Hurd YL Preferential limbic expression of the cannabinoid receptor mRNA in the human fetal brain Neuroscience 2003 118 3 681 694 10.1016/S0306-4522(03)00020-4 12710976

[B63] Burgunder JM Young WS Cortical neurons expressing the cholecystokinin gene in the rat: distribution in the adult brain, ontogeny, and some of their projections J Comp Neurol 1990 300 1 26 46 10.1002/cne.903000104 2229486

[B64] Hökfelt T Morino P Vergeb V Castel MN Broberger C Zhang X Herrera-Marschitz M Meana JJ Ungerstedt U Xu XJ CCK in Cerebral Cortex and at the Spinal Level Ann NY Acad Sci 1994 713 1 157 163 10.1111/j.1749-6632.1994.tb44062.x 8185156

[B65] Hayes TL Lewis DA Nonphosphorylated neurofilament protein and calbindin immunoreactivity in layer III pyramidal neurons of human neocortex Cereb Cortex 1992 2 1 56 67 10.1093/cercor/2.1.56 1633408

[B66] Kang HJ Kawasawa YI Cheng F Zhu Y Xu X Li M Sousa AM Pletikos M Meyer KA Sedmak G Spatio-temporal transcriptome of the human brain Nature 2011 478 7370 483 489 10.1038/nature10523 22031440 PMC3566780

[B67] Eggan SM Mizoguchi Y Stoyak SR Lewis DA Development of cannabinoid 1 receptor protein and messenger RNA in monkey dorsolateral prefrontal cortex Cereb Cortex 2010 20 5 1164 1174 10.1093/cercor/bhp179 19703937 PMC2852503

[B68] Diamond A Development of the ability to use recall to guide action, as indicated by infants' performance on AB Child Dev 1985 56 4 868 883 10.2307/1130099 4042750

[B69] Berrendero F Sepe N Ramos JA Di Marzo V Fernandez-Ruiz JJ Analysis of cannabinoid receptor binding and mRNA expression and endogenous cannabinoid contents in the developing rat brain during late gestation and early postnatal period Synapse 1999 33 3 181 191 10.1002/(SICI)1098-2396(19990901)33:3<181::AID-SYN3>3.0.CO;2-R 10420166

[B70] Morishita J Okamoto Y Tsuboi K Ueno M Sakamoto H Maekawa N Ueda N Regional distribution and age-dependent expression of N-acylphosphatidylethanolamine-hydrolyzing phospholipase D in rat brain J Neurochem 2005 94 3 753 762 10.1111/j.1471-4159.2005.03234.x 15992380

[B71] Chavez AE Chiu CQ Castillo PE TRPV1 activation by endogenous anandamide triggers postsynaptic long-term depression in dentate gyrus Nat Neurosci 2010 13 12 1511 1518 10.1038/nn.2684 21076423 PMC3058928

[B72] Grueter BA Brasnjo G Malenka RC Postsynaptic TRPV1 triggers cell type-specific long-term depression in the nucleus accumbens Nat Neurosci 2010 13 12 1519 1525 10.1038/nn.2685 21076424 PMC3092590

[B73] Maione S Cristino L Migliozzi AL Georgiou AL Starowicz K Salt TE Di Marzo V TRPV1 channels control synaptic plasticity in the developing superior colliculus J Physiol 2009 587 Pt 11 2521 2535 19406878 10.1113/jphysiol.2009.171900 PMC2714018

[B74] Liapi A Wood JN Extensive co-localization and heteromultimer formation of the vanilloid receptor-like protein TRPV2 and the capsaicin receptor TRPV1 in the adult rat cerebral cortex Eur J Neurosci 2005 22 4 825 834 10.1111/j.1460-9568.2005.04270.x 16115206

[B75] Maccarrone M Rossi S Bari M De Chiara V Fezza F Musella A Gasperi V Prosperetti C Bernardi G Finazzi-Agro A Anandamide inhibits metabolism and physiological actions of 2-arachidonoylglycerol in the striatum Nat Neurosci 2008 11 2 152 159 10.1038/nn2042 18204441

[B76] Volk DW Austin MC Pierri JN Sampson AR Lewis DA Decreased glutamic acid decarboxylase67 messenger RNA expression in a subset of prefrontal cortical gamma-aminobutyric acid neurons in subjects with schizophrenia Arch Gen Psychiatry 2000 57 3 237 245 10.1001/archpsyc.57.3.237 10711910

[B77] Lewis DA Gonzalez-Burgos G Neuroplasticity of neocortical circuits in schizophrenia Neuropsychopharmacology 2007 33 1 141 165 17805309 10.1038/sj.npp.1301563

[B78] Yizhar O Fenno LE Prigge M Schneider F Davidson TJ O/'Shea DJ Sohal VS Goshen I Finkelstein J Paz JT Neocortical excitation/inhibition balance in information processing and social dysfunction Nature 2011 advance online publication 10.1038/nature10360 PMC4155501 21796121

[B79] Hajós M Hoffmann WE Kocsis B Activation of cannabinoid-1 receptors disrupts sensory gating and neuronal oscillation: relevance to schizophrenia Biol Psychiatry 2008 63 11 1075 1083 10.1016/j.biopsych.2007.12.005 18261715

[B80] Hajos N Katona I Naiem SS MacKie K Ledent C Mody I Freund TF Cannabinoids inhibit hippocampal GABAergic transmission and network oscillations Eur J Neurosci 2000 12 9 3239 3249 10.1046/j.1460-9568.2000.00217.x 10998107

[B81] Wong J Webster MJ Cassano H Weickert CS Changes in alternative brain-derived neurotrophic factor transcript expression in the developing human prefrontal cortex Eur J Neurosci 2009 29 7 1311 1322 10.1111/j.1460-9568.2009.06669.x 19519623

[B82] Weickert CS Elashoff M Richards AB Sinclair D Bahn S Paabo S Khaitovich P Webster MJ Transcriptome analysis of male–female differences in prefrontal cortical development Mol Psychiatry 2009 14 6 558 561 10.1038/mp.2009.5 19455171

[B83] Kozlovsky N Shannon Weickert C, Tomaskovic-Crook E, Kleinman JE, Belmaker RH, Agam G: Reduced GSK-3ß mRNA levels in postmortem dorsolateral prefrontal cortex of schizophrenic patients J Neural Transm 2004 111 12 1583 1592 10.1007/s00702-004-0166-3 15565492

[B84] Mimmack ML Ryan M Baba H Navarro-Ruiz J Iritani S Faull RLM McKenna PJ Jones PB Arai H Starkey M Gene expression analysis in schizophrenia: reproducible up-regulation of several members of the apolipoprotein L family located in a high-susceptibility locus for schizophrenia on chromosome 22 Proc Natl Acad Sci U S A 2002 99 7 4680 4685 10.1073/pnas.032069099 11930015 PMC123707

[B85] Whitfield HJ Brady LS Smith MA Mamalaki E Fox RJ Herkenham M Optimization of cRNA probe in situ hybridization methodology for localization of glucocorticoid receptor mRNA in rat brain: a detailed protocol Cell Mol Neurobiol 1990 10 1 145 157 10.1007/BF00733641 2334945 PMC11567182

[B86] Romanczyk TB Weickert CS Webster MJ Herman MM Akil M Kleinman JE Alterations in trkB mRNA in the human prefrontal cortex throughout the lifespan Eur J Neurosci 2002 15 2 269 280 10.1046/j.0953-816x.2001.01858.x 11849294

[B87] Rajkowska G Goldman-Rakic PS Cytoarchitectonic definition of prefrontal areas in the normal human cortex: II. Variability in locations of areas 9 and 46 and relationship to the Talairach Coordinate System Cereb Cortex 1995 5 4 323 337 10.1093/cercor/5.4.323 7580125

