# Supplementary figures and images for: Developmental trajectory of the endocannabinoid system in human dorsolateral prefrontal cortex
Source: BMC Neurosci. 2012 Jul 24;13:87. doi: 10.1186/1471-2202-13-87 (PMC3464170; doi:10.1186/1471-2202-13-87)

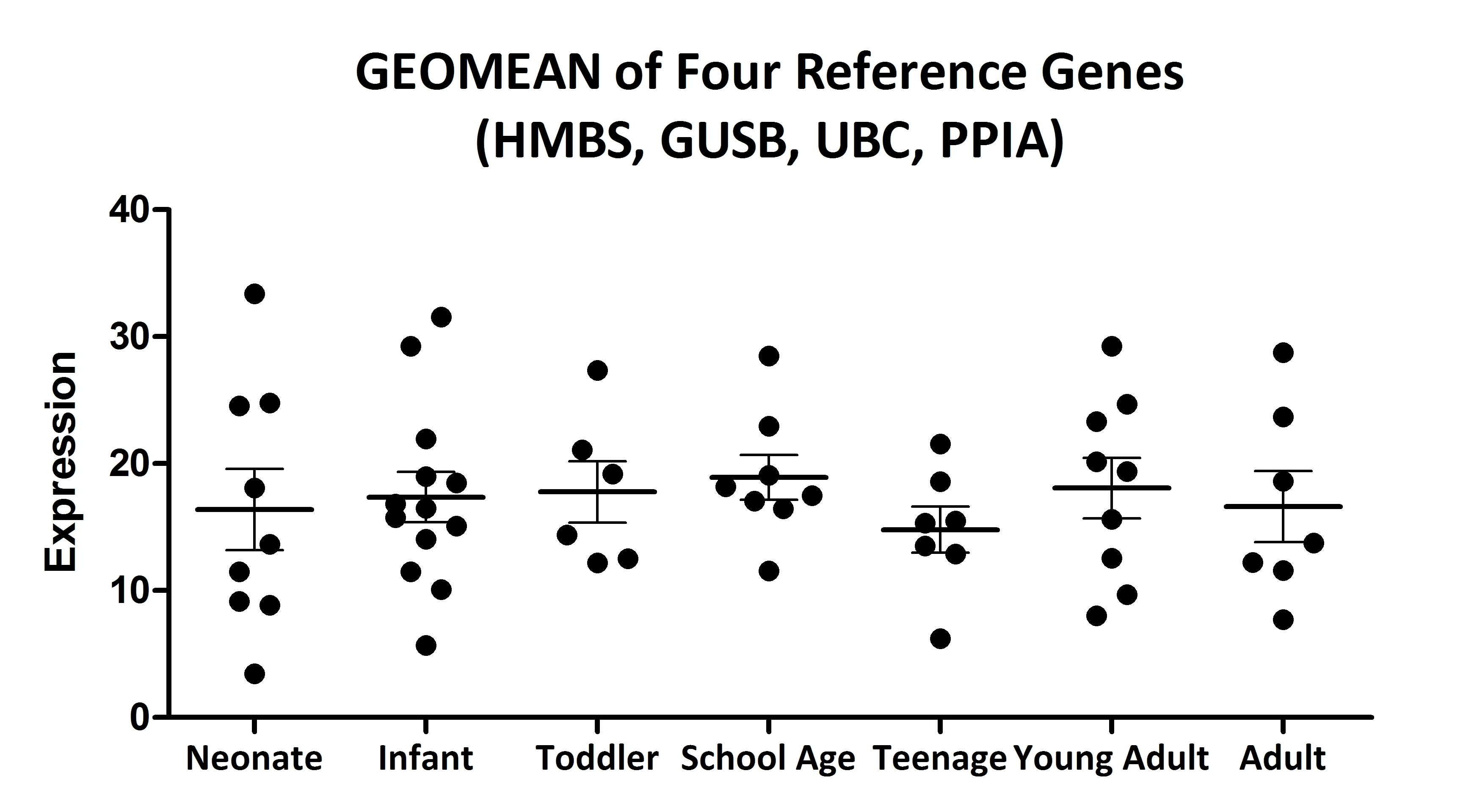

Supplement: Additional file 1 — Figure S1. Geometric mean of expression of HMBS, GUSB, UBC and PPIA mRNA in human DLPFC determined by qPCR [y-axis, mean (+ S.E.M.)] expression plotted by age group. n = 7 – 13. [file 1471-2202-13-87-S1.tiff]

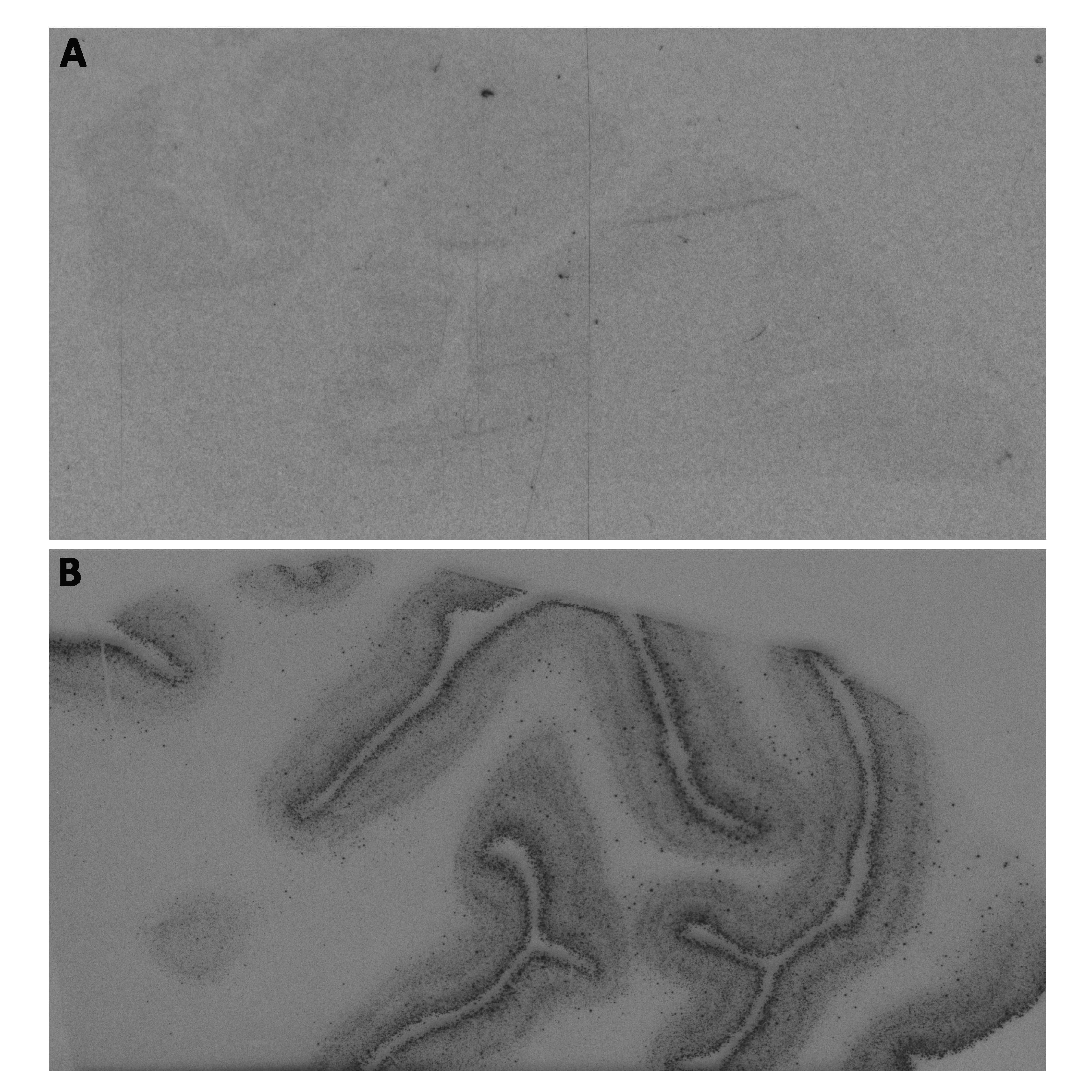

Supplement: Additional file 2 — Figure S2. Representative autoradiograms from sections from two school-aged individuals showing A CB1R mRNA hybridisation after antisense strand riboprobe incubation and Bnegligible CB1R mRNA hybridisation after sense strand riboprobe incubation. [file 1471-2202-13-87-S2.tiff]
